# Supplementary material for: Crystal structure of a highly conserved enteroviral 5′ cloverleaf RNA replication element
Source: Nat Commun. 2023 Apr 7;14:1955. doi: 10.1038/s41467-023-37658-8 (PMC10082201; doi:10.1038/s41467-023-37658-8)
Supplement: Supplementary file 1 — Supplementary Information [file 41467_2023_37658_MOESM1_ESM.pdf]

Supplementary information

Crystal structure of a highly conserved enteroviral 5′ cloverleaf RNA replication element

Naba K. Das,[1] Nele M. Hollmann,[1,2] Jeff Vogt,[1] Spiridon E. Sevdalis,[3] Hasan A. Banna,[1] Manju Ojha,[1] Deepak Koirala[1,*]

[1]Department of Chemistry and Biochemistry, University of Maryland Baltimore County, Baltimore, Maryland 21250, USA

[2]Howard Hughes Medical Institute, University of Maryland Baltimore County, Baltimore, Maryland 21250, USA

[3]Department of Biochemistry and Molecular Biology, University of Maryland School of Medicine, Baltimore, MD 21201, USA

*Corresponding author Email: dkoirala@umbc.edu

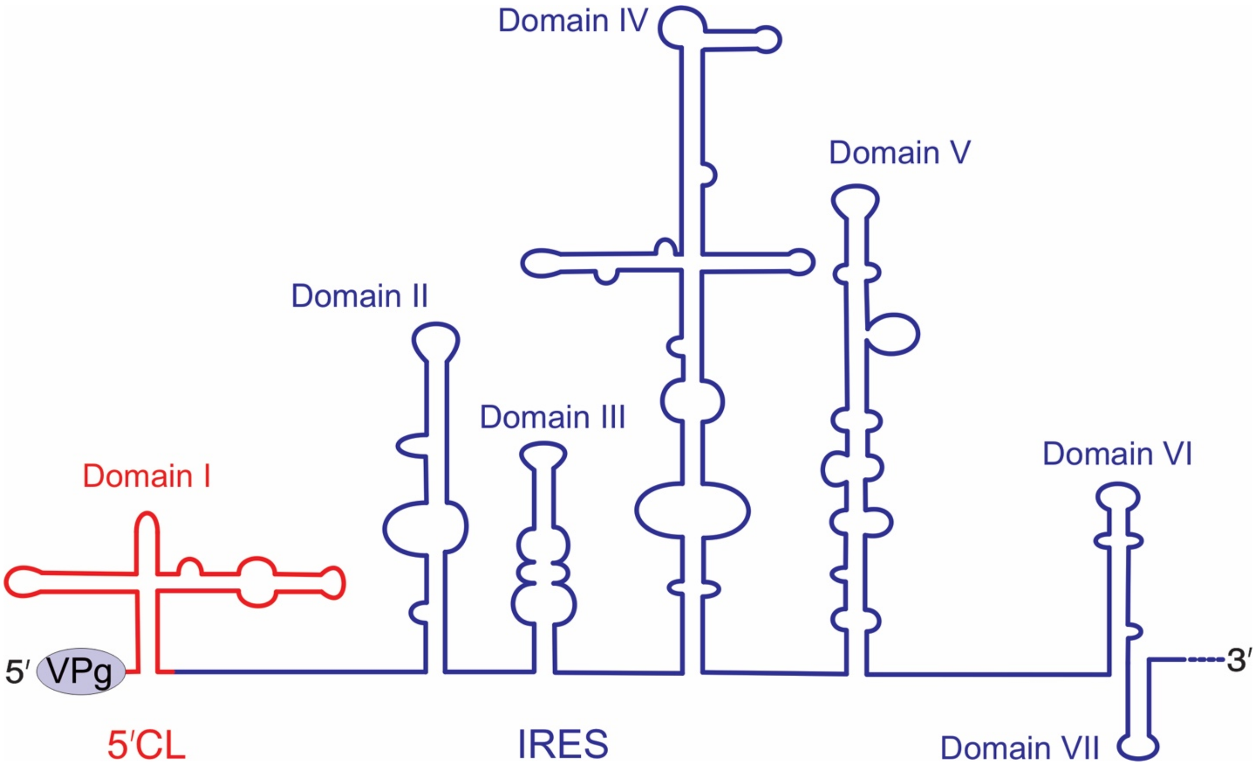

Supplementary Figure 1. Schematic illustration of the modular RNA domains within the 5′ UTR of enteroviral genomes. Shown here is the secondary structural model of CVB3 according to Bailey *et al*.[1] Domain I (colored red) represents the cloverleaf-like (5′CL) domain, and domains II to VII (colored blue) comprise the internal ribosome entry site (IRES).

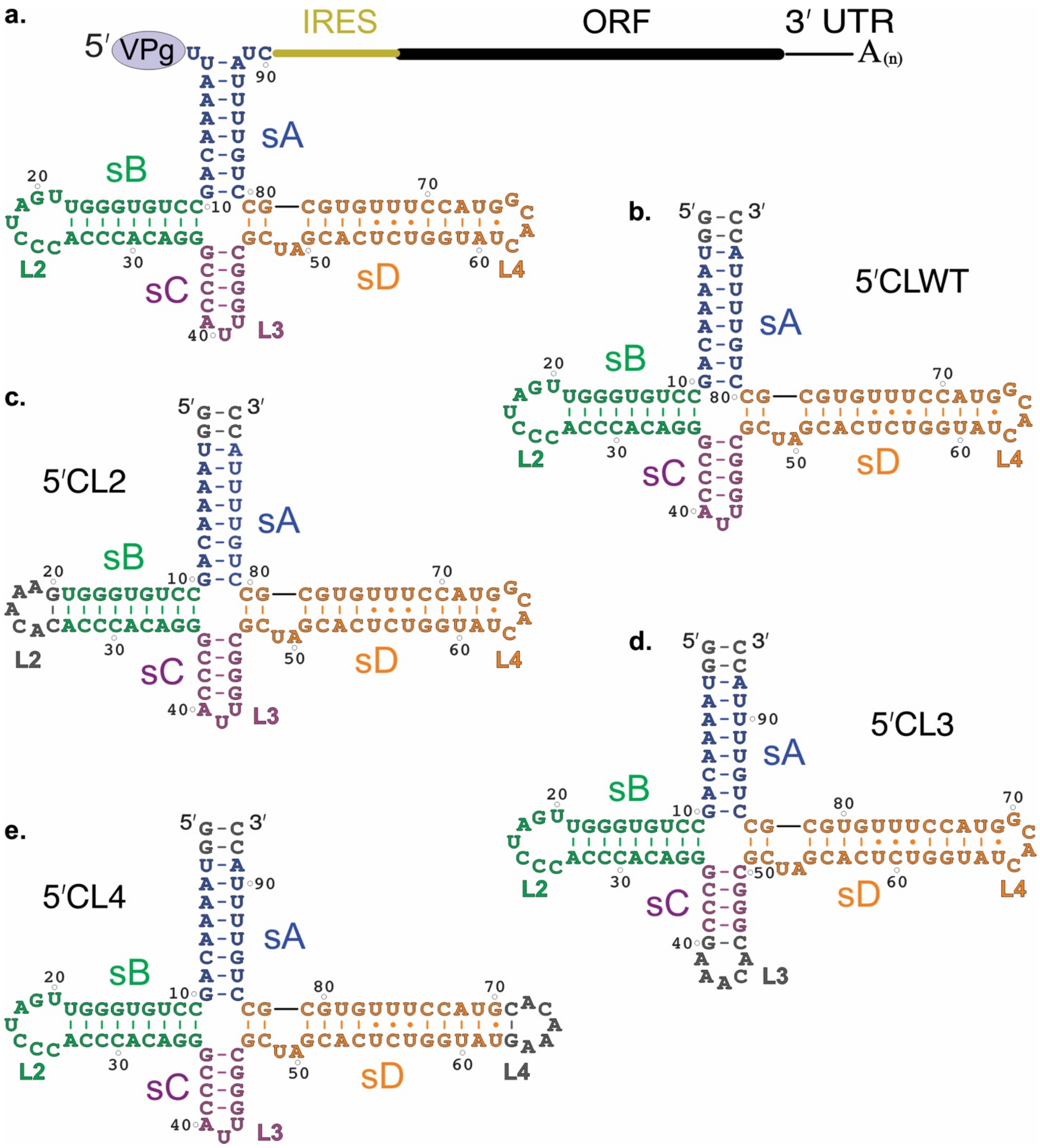

Supplementary Figure 2. The 5′CL RNA crystallization constructs. (a) The proposed secondary structure of CVB3 5′CL within the 5′ UTR of the CVB3 genome. The wild-type crystallization construct (b) includes the two G-C base pairs (colored gray) closing the sA helix. The 5′CL2 (c), 5′CL3 (d), and 5′CL4 (e) are the crystallization constructs with the sB, sC, and sD loops replaced by a Fab BL3-6 binding motif (5′-GAAACAC sequence, colored gray), respectively.

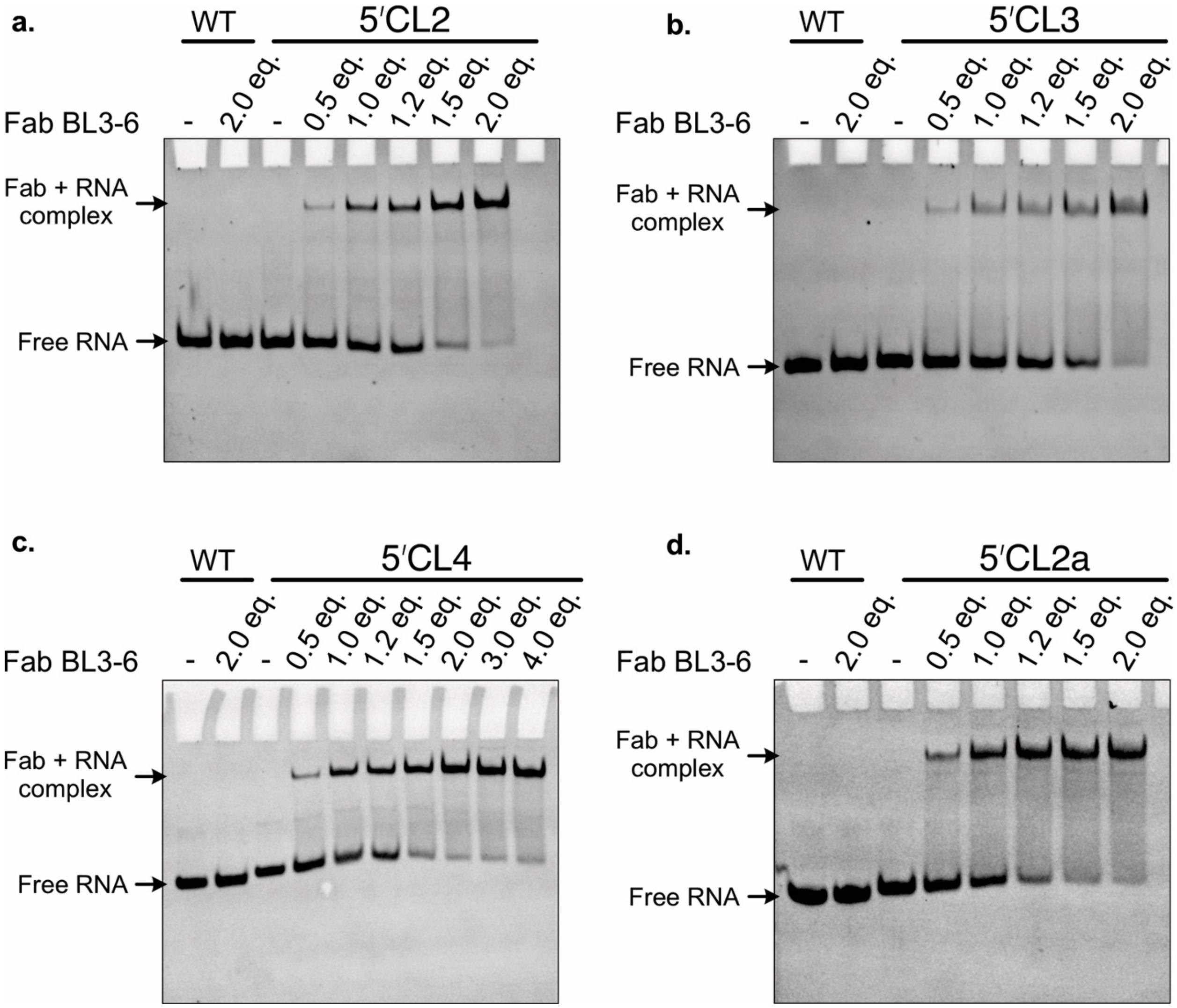

Supplementary Figure 3. The native polyacrylamide gel electrophoresis (nPAGE) to test the binding of Fab BL3-6 with the crystallization RNA constructs (see Methods for the experimental details). Each lane contains 100 ng of RNA. The wild-type (without Fab binding motif) serves as a negative control. The slower migrating Fab-RNA complex bands appear only for the constructs with the Fab binding motif.

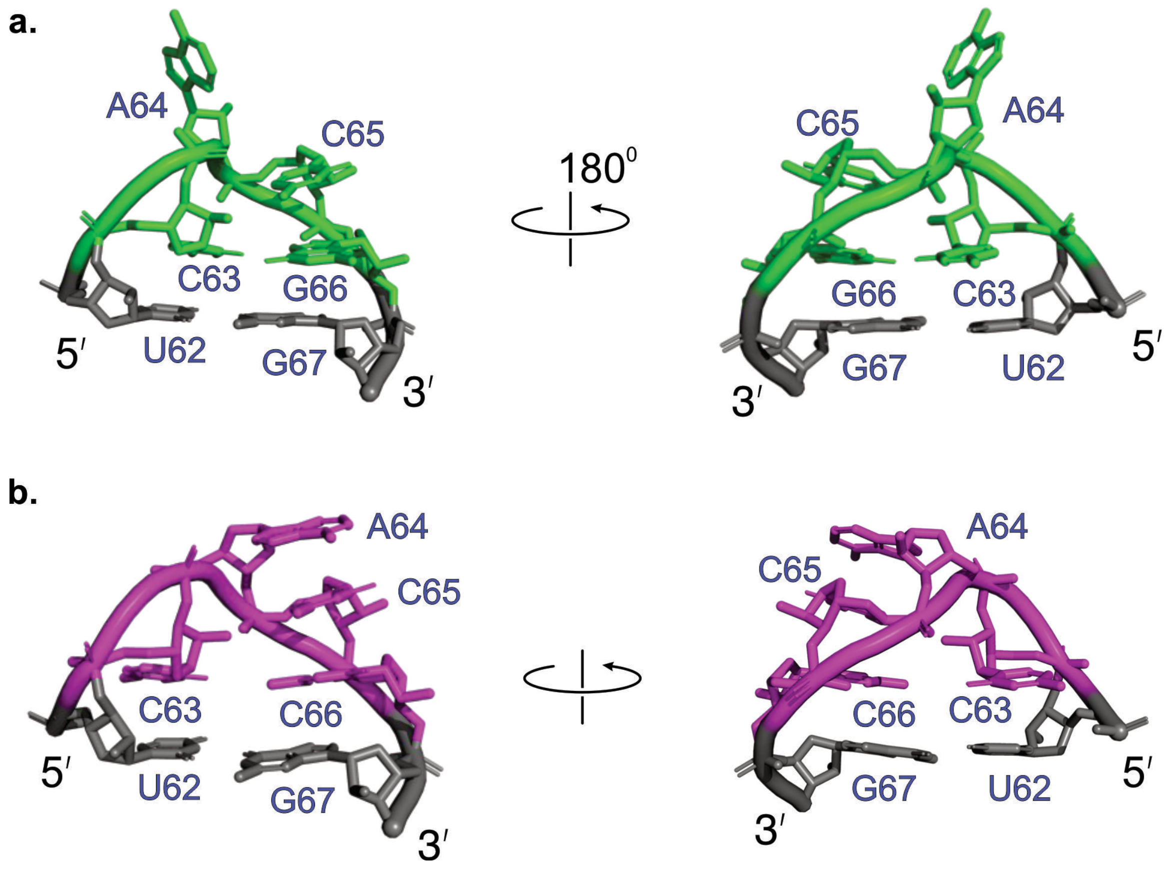

Supplementary Figure 4. Models of the sD tetraloops for (a) uCACGg (wild-type) and (b) uCACCg (crystallized construct) as predicted by the ROSIE (Rosetta Online Server that Includes Everyone, https://rosie.rosettacommons.org/rna_denovo).[2, 3] The wild-type sequence folded into a UNCG-type tetraloop, consistent with the previous in-solution NMR results,[4-6] whereas the mutant folded into a GNRA-type tetraloop similar to that observed in the wild-type UNCG-type tetra-loop crystal structure. Noticeably, nucleotide A64 that facilitated the crystal contacts in the crystallized loop (see Supplementary Figure 8 below) appeared extended out in the wild-type loop that would have clashed with symmetry-related Fabs, which perhaps explains the low-resolution X-ray diffraction of the 5′CL2 construct with uCACGg loop compared to the mutant, 5′CL2a construct with the uCACCg loop.

Supplementary Table 1. X-ray crystallography data collection and structure refinement statistics. Values in the parentheses are for the highest resolution shell.

**Data collection**	
Space group	C 1 2 1
Resolution (Å)	66.62 – 1.91 (1.98 – 1.91)
Cell dimensions	
a, b, c (Å)<br>α, β, γ (°)	122.20, 48.70, 144.65<br>90, 112.92, 90
R_{merge} (%)	8.2 (88.7)
I/σI	10.3 (1.8)
$CC_{1/2}$	0.998 (0.744)
Completeness (%)	97.6 (97.9)
Redundancy	3.3 (3.3)
**Refinement**	
No. reflections	54, 094 (3739)
$R_{work} – R_{free}$ (%)	20.9 – 25.9
R.M.S. deviations	
Bond angles (°)	0.950
Bond length (Å)	0.006
Average B-factor, all atoms ($Å^2$)	55.48
Ramachandran plot of protein residues	
Preferred regions (%)	98.40
Allowed regions (%)	1.60

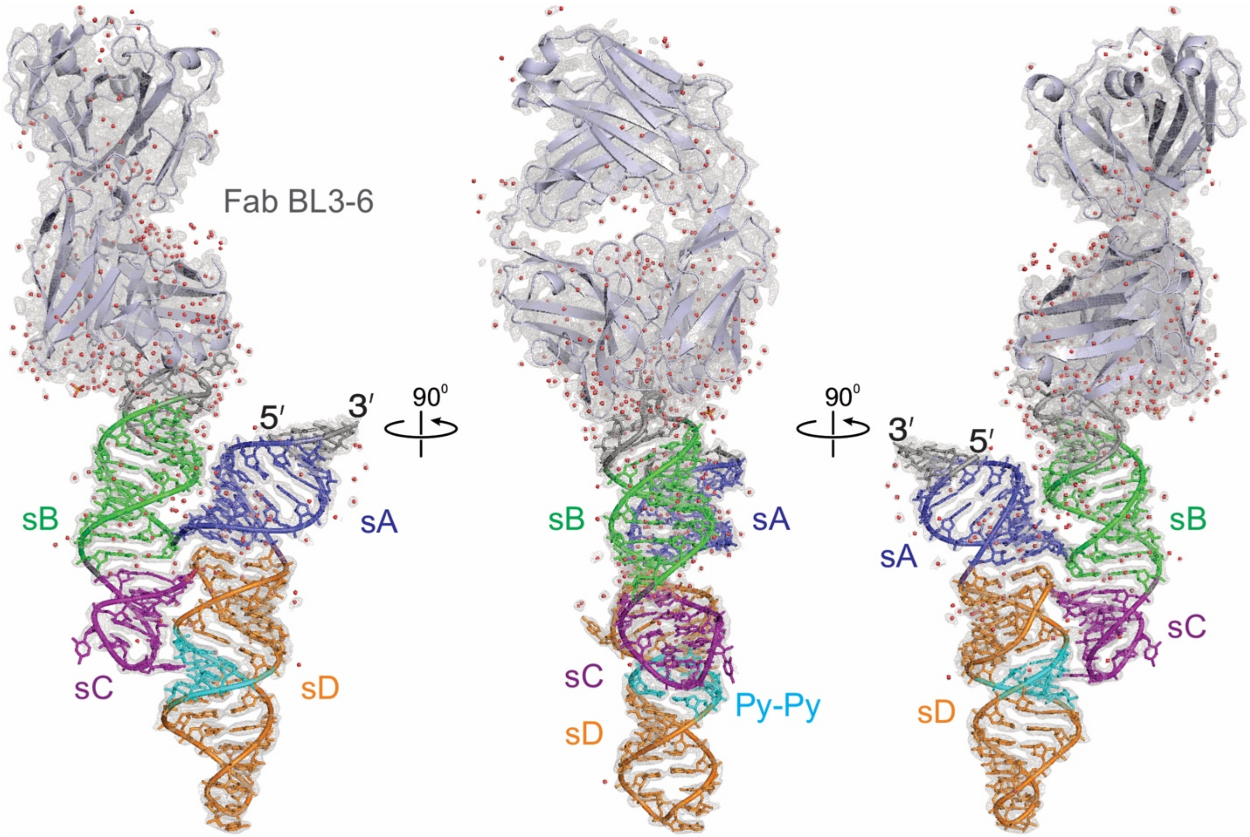

Supplementary Figure 5. Overall crystal structure of the CVB3-5′CL2a in complex with Fab BL3-6 solved at 1.9 Å resolution. The crystals had a single Fab-RNA complex per asymmetric unit. The red spheres depict water molecules, and the gray mesh represents the $2|F_o| - |F_c|$ electron density map at 1σ contour level and carve radius 1.8 Å.

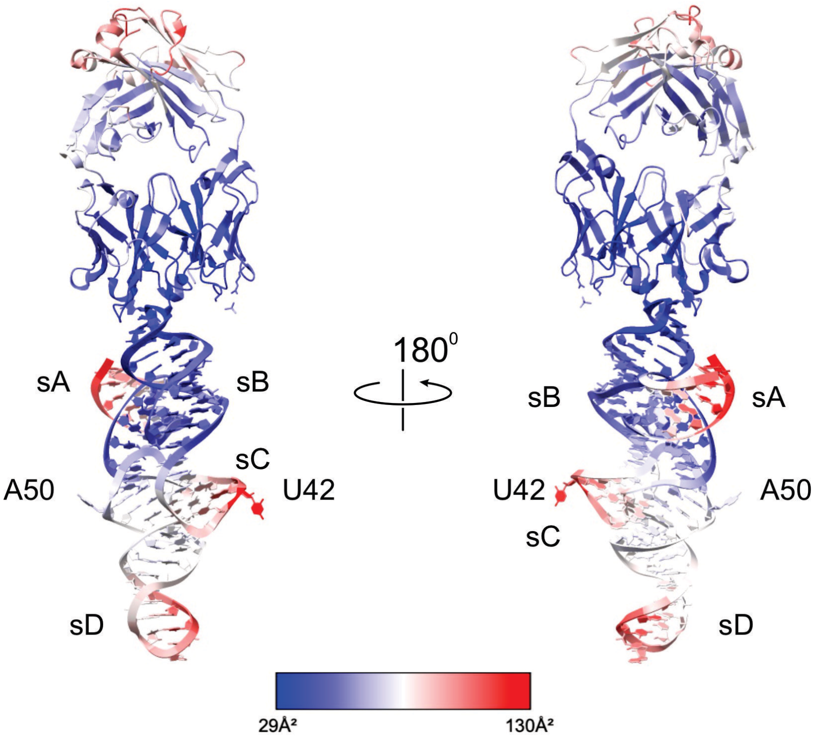

Supplementary Figure 6. The crystal structure of the 5′CL2a in complex with Fab BL3-6 colored according to the crystallographic B-factors. The gradient from blue to red indicates the lowest (29 Å2) and the highest (130 Å2) B-factors for the structure.

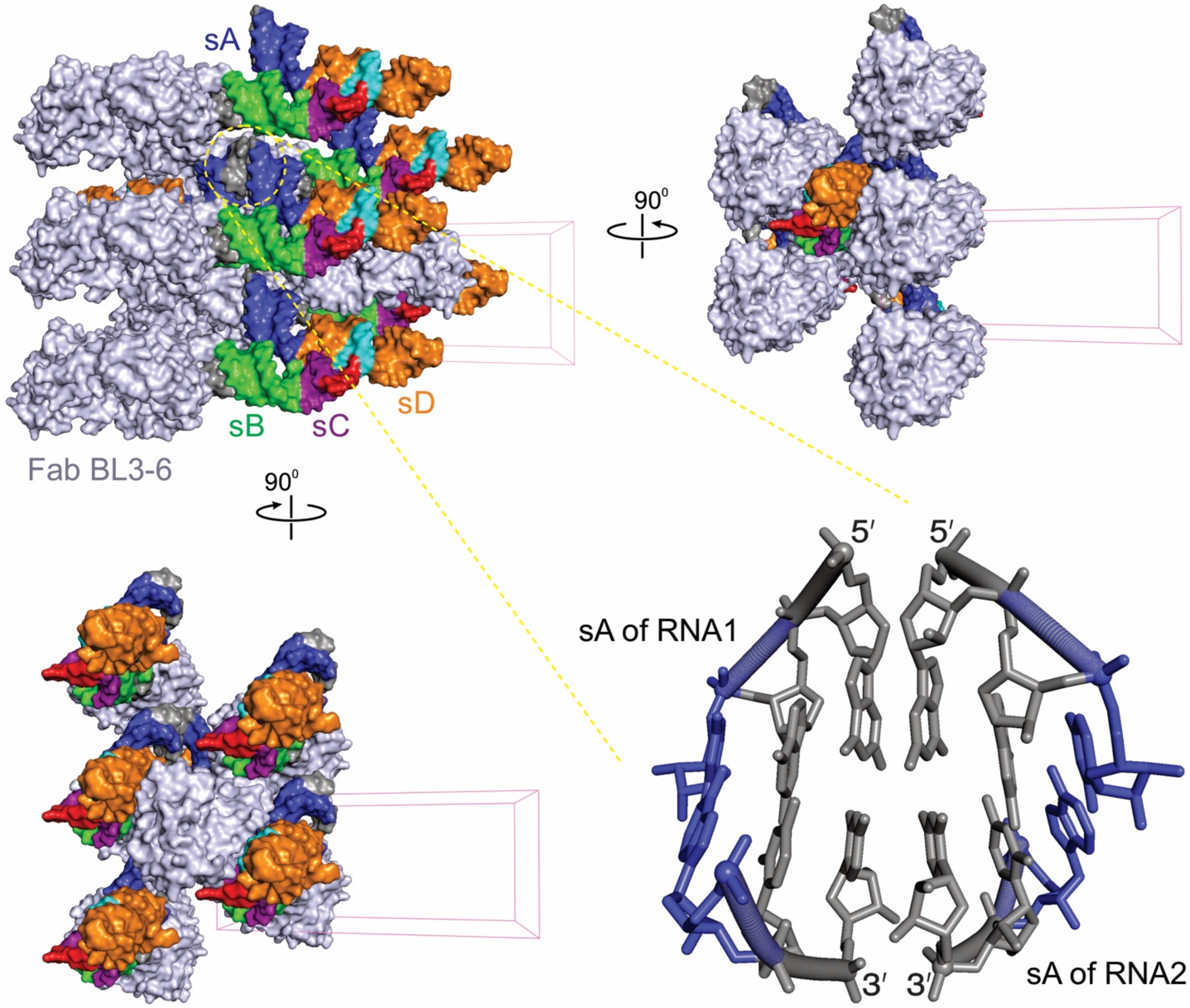

Supplementary Figure 7. The RNA and Fab-mediated crystal contacts within the crystallographic asymmetric unit. Within the crystal lattice of the 5′CL2a-BL3-6 complex, including the Fab-RNA binding interface, the Fab interactions account for about 92% of the buried surface area, suggesting a critical role of the Fab in the crystallization. The RNA-RNA interactions involve only the end-to-end stacking of the sA helices from symmetry-related RNA molecules, accounting for about 8% of the buried surface area.

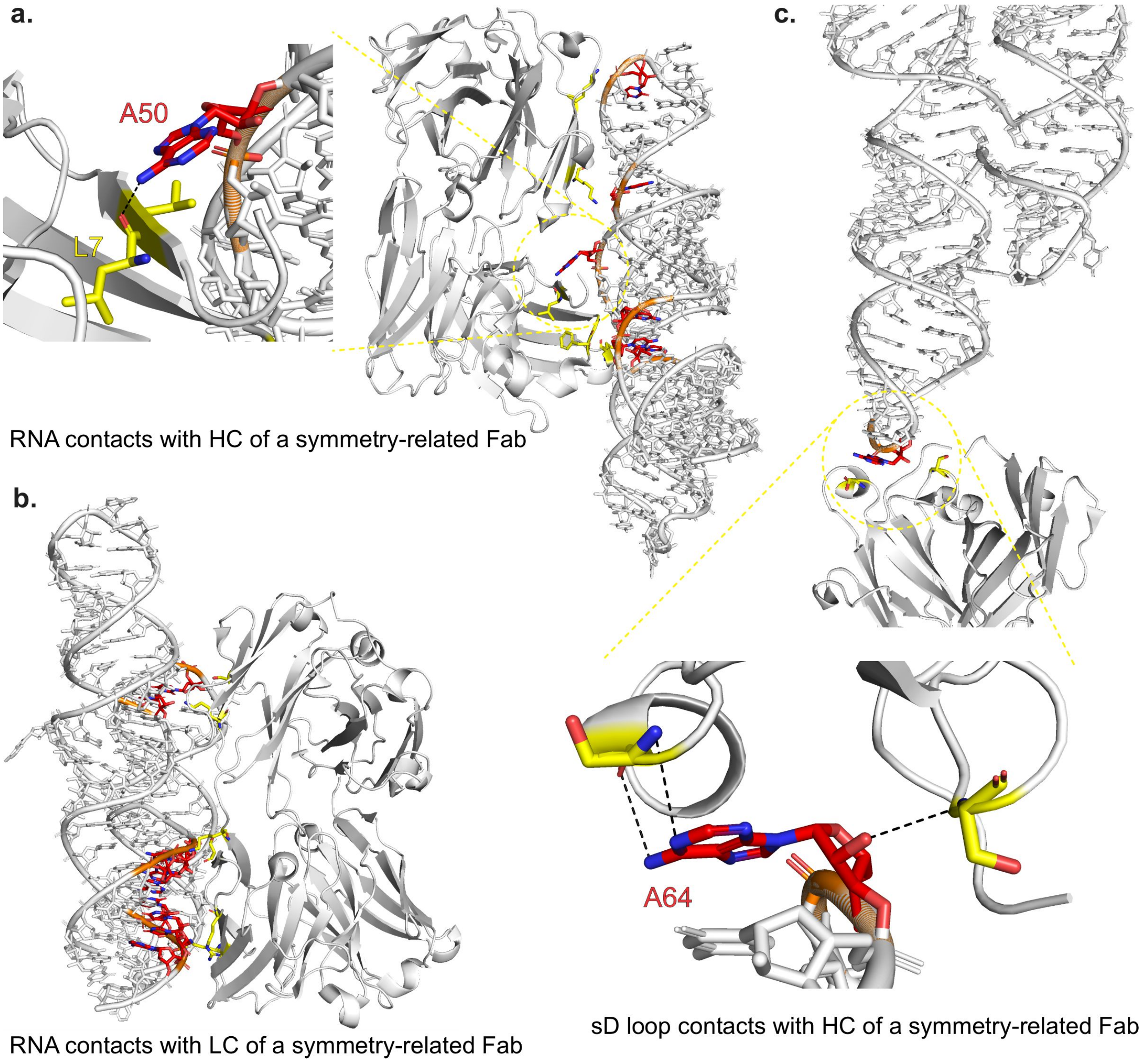

Supplementary Figure 8. The Fab-RNA contacts as observed in the crystals of CVB3 5′CL2a RNA in complex with Fab BL3-6. The scaffold residues from the (a) heavy chain (HC) and (b) the light chain (LC) of the symmetry-related Fab make contact with the RNA. Especially the side chains of positively charged residues, such as lysines and arginines, interact with the RNA backbone. The A50 nucleobase within the sD bulge (a) and A64 nucleotide within the sD tetraloop (d) also make contact with the heavy chain residues of the symmetry-related Fab molecules. The Fab residues and the RNA regions involved in the crystal contacts are highlighted in yellow and red, respectively. The dashed lines represent the hydrogen bonding distances.

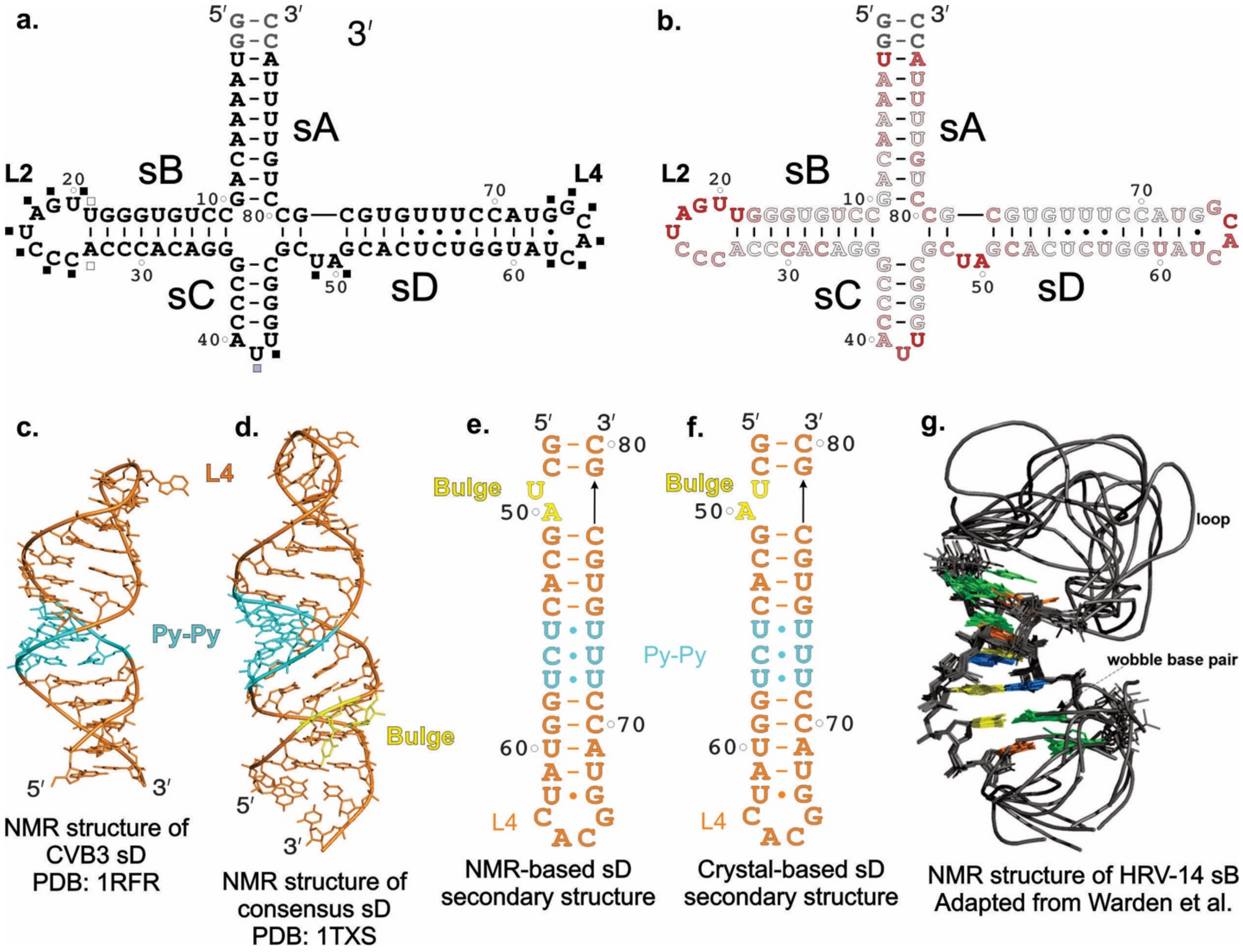

Supplementary Figure 9. Comparison of previous biochemical probing results and NMR-based structures with the CVB3 5′CL crystal structure. (a) The secondary structure of CVB3 5′CL shows the modified positions (filled squares) by Kethoxal, DMS, or CMCT according to the data taken from Bailey et al.[1] and Prusa et al.[7] The black and gray squares identify strongly and moderately modified positions, respectively. (b) The same CVB3 5′CL structure shows the SHAPE reactivity at each position, as indicated by the white-to-red color, according to data retrieved from Mahmud et al.[8] The nucleotides colored white and dark red refer to the lowest and highest SHAPE reactivity, respectively. The pink-colored nucleotides represent intermediate SHAPE reactivity. Both biochemical probing and SHAPE data shown here were performed in the context of the intact 5′UTR. (c) NMR structure of CVB3 sD (without the bulge portion, PDB: 1RFR),[6] and (d) a consensus sD (including the bulge portion, PDB: 1TXS).[5] Except for the flipped nucleotide (A or U) in the bulge, the NMR-derived secondary structure of the isolated CVB3 sD (e) is identical to that derived from the crystal structure of intact 5′CL (f). The NMR structure of HRV-14 sB (g)

taken from a publication by Warden et al.[9] shows no unpaired nucleotides within the sB helix. Overall, the secondary structural features of the enteroviral 5′CL observed in the solution by biochemical probing, SHAPE, and NMR agree with those observed in the crystal, supporting that the crystal structure of CVB3 5′CL represents that in the solution.

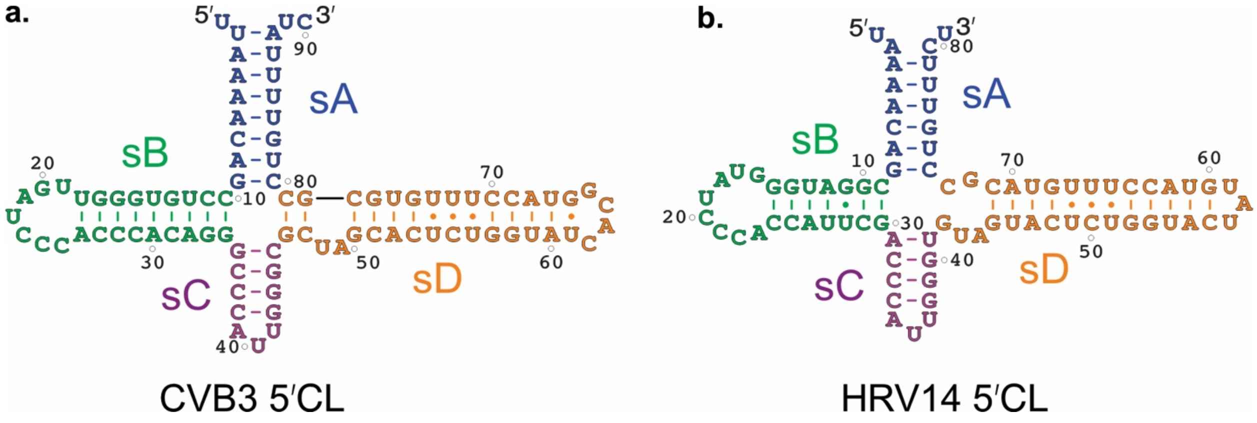

Supplementary Figure 10. Comparison of the CVB3 and HRV 5′CLs. The predicted secondary structures of the CVB3 (a) and the HRVB14 (b) 5′CLs. The subdomains and labels are colored analogously for facile comparison.

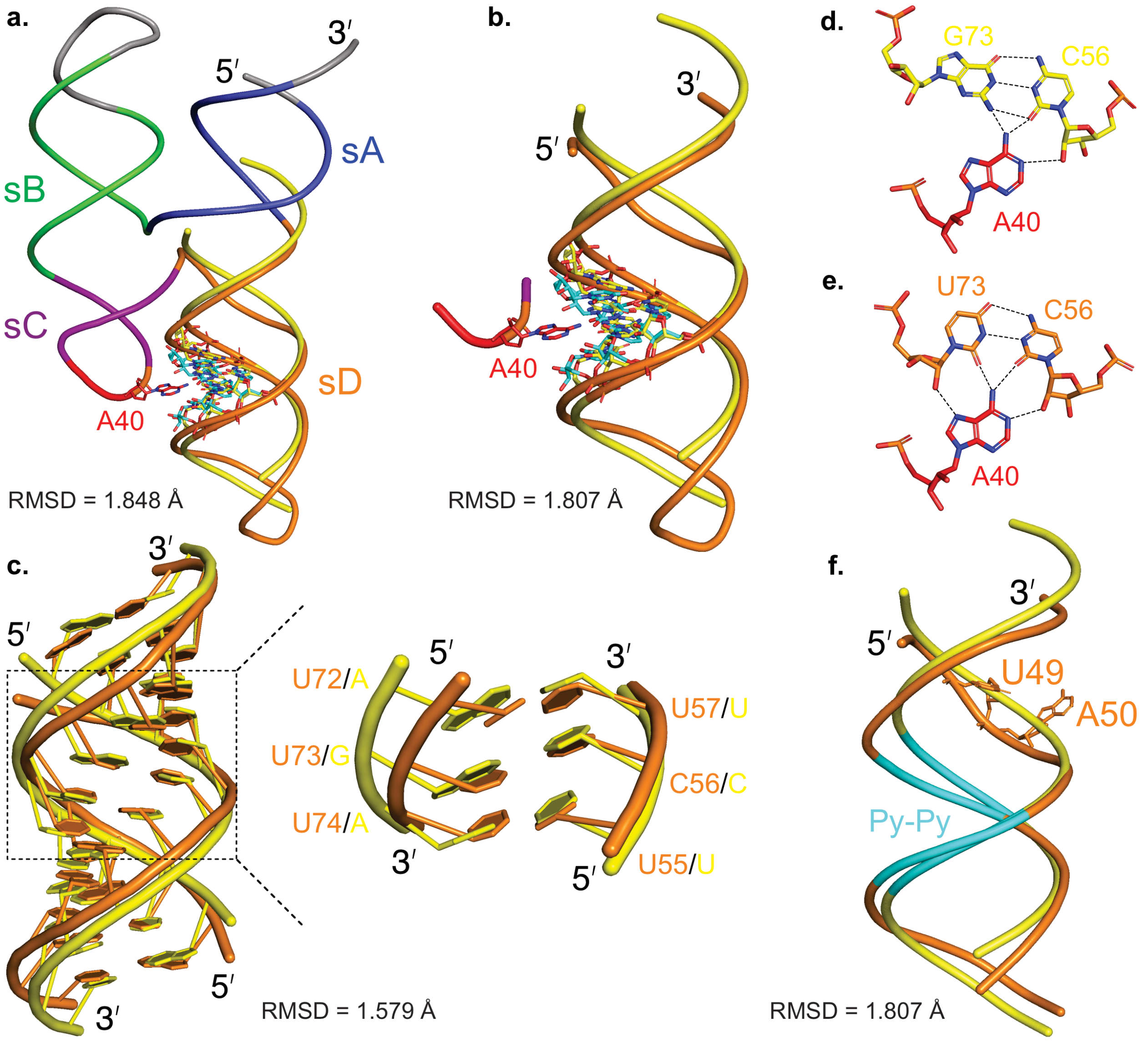

Supplementary Figure 11. Comparison of the sD subdomain structural features with an ideal A-form RNA helix. (a) Superposition of an ideal A-form helix (yellow) with the sD subdomain (orange) of the intact 5′CL crystal structure (RMSD = 1.848 Å). For simplistic comparison, the ideal A-form helix was created computationally for the same sD sequence, G47 to U62, excluding the loop L4. (b) Superposition of the ideal A-form helix (yellow) with the isolated sD subdomain (orange) without the loop L4 (RMSD = 1.807 Å). As observed in the crystal structure, the A40 interaction with the sD helix is also shown for comparison. Except for the slight widening of the ideal helix compared to the Py-Py helix, perhaps due to the larger purine bases in the ideal helix compared to all pyrimidine bases in the Py-Py helix (c), the sD helix after the bulge showed no significant deviation from ideal A-form helix (RMSD = 1.579 Å). Additionally, the presence of

the Py-Py mismatch pair in the middle of the sD helix seems not to change the relative orientations of the sD helix ends (a-c). Interestingly, the replacement of the Py-Py region by canonical base pairs still allows A40-sD helix interactions (d) in a similar fashion as observed in the crystal structure (e), which is consistent with previous observations that these changes did not inhibit the (-)-strand synthesis.[10, 11] Perhaps the A40-sD helix interactions are to correctly position the 3C and PCBP binding sites at the opposite ends of the 5′CL structure, which is consistent with previous observations that any deletions in the Py-Py region, which likely bend the sD helix in the middle changing the relative positions of 3C and PCBP binding sites, inhibits the (-)-strand synthesis. Absolute conservation of the Py-Py region may be required for the back-synthesis of the (+)-strand using the (-)-strand as the template, where a similar cloverleaf structure (3′CL) is likely to form but with the complementary sequence, consistent with the ability of 3′CL to bind a different set of proteins compared to the 5′CL.[12-16] Moreover, although Py-Py non-canonical sD helix compared to the corresponding ideal A-form helix preserved the orientation of the ends and interactions with the A40, the presence of the bulge at the base of the sD helix seems to shorten the length of the sD helix between the four-way junction and the Py-Py region (f). The arrangement helps precisely position the central C56•U73 pair of the Py-Py helix for interactions with the A40, consistent with the previous observations that the sD bulge is essential for the (-)-strand synthesis.[10, 11]

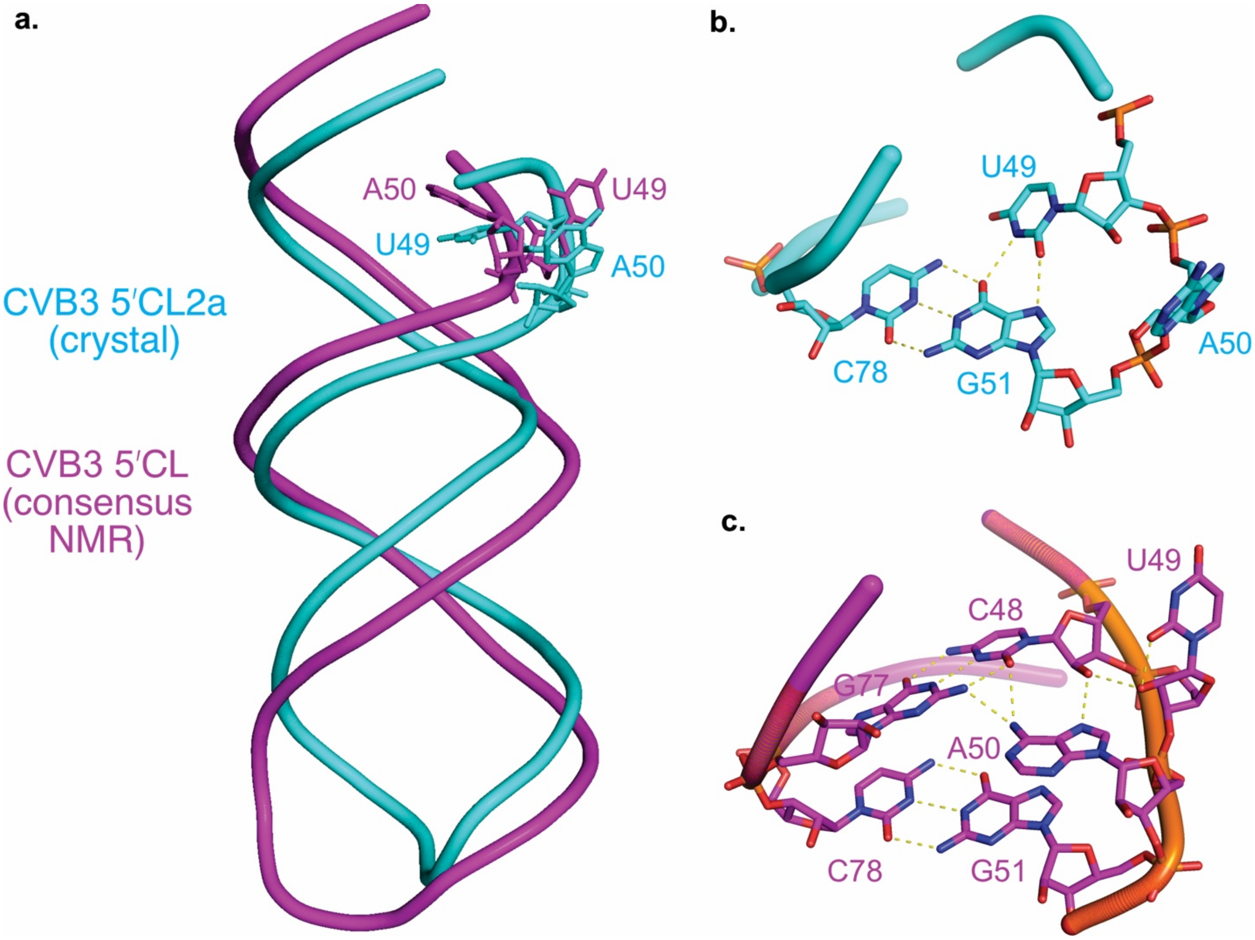

Supplementary Figure 12. Structural features of the dinucleotide bulge within the sD subdomain of enteroviral 5′CLs. (a) Superposition of the sD subdomains from our crystal structure of CVB3 5′CL (cyan) and the NMR structure of a consensus enteroviral sequence (magenta).[5] The structures superimpose well (overall RMSD = 3.461 Å, without the bulge and tetraloop = 3.290 Å) except for the tetraloop and dinucleotide bulge regions. Within the bulge, the crystal structure shows the flipped-out A50 and helically stacked U49 (b), whereas the consensus structure shows the flipped-out U49 and helically stacked A50 (c). In the former structure, the base-triple U49•G51-C78 stabilizes the bulge, but in the latter configuration, a network of hydrogen bonds involving C48, U49, A50, and G77, including a potential A50•C48 G77 base-triple. Both confirmations may exist in the solution. However, a strong hydrogen bonding network within the bulge supports a stable rather than a dynamic configuration of this bulge. It is also noteworthy that the NMR structure represents an isolated sD only. Subdomains and labels are colored analogously for facile comparison.

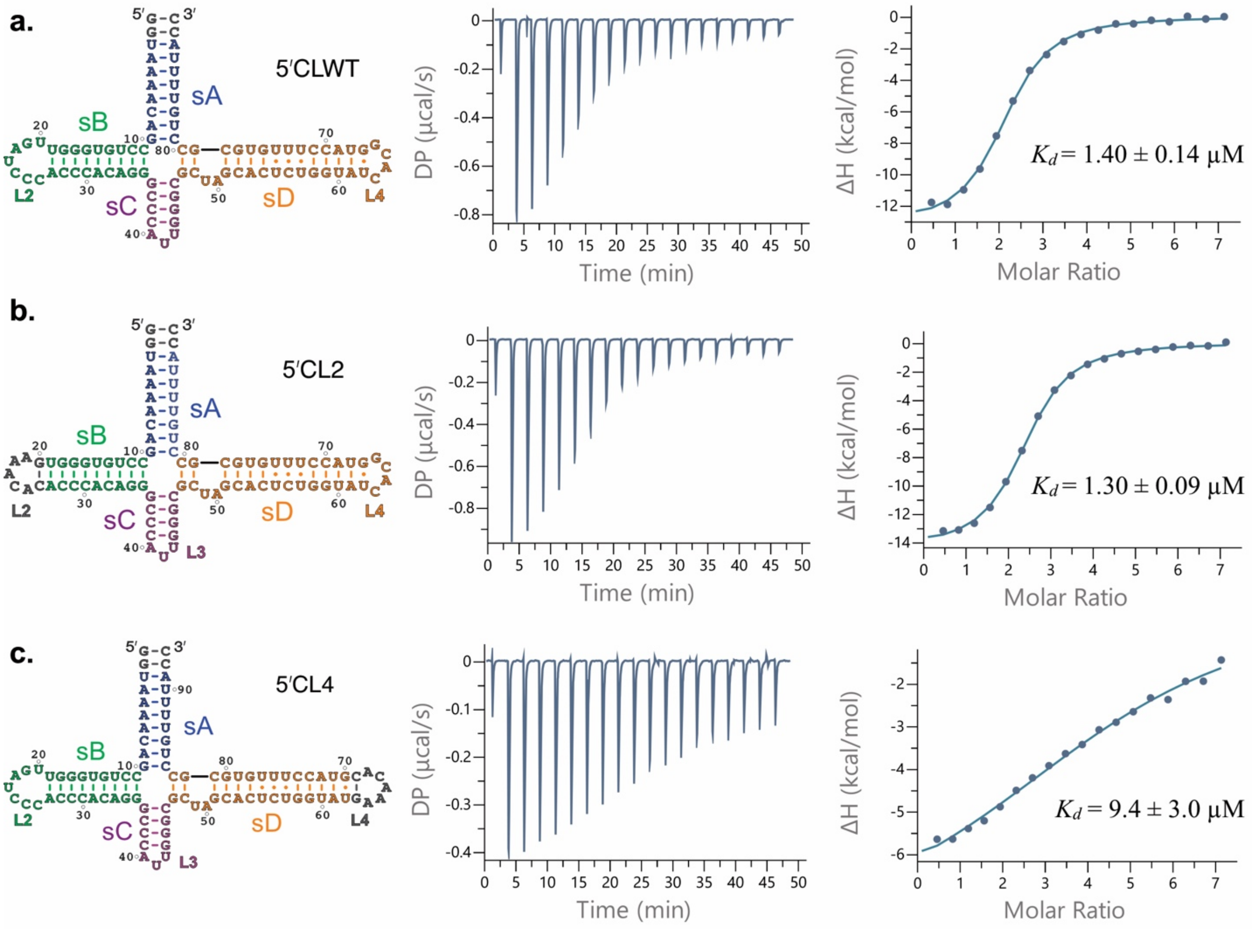

Supplementary Figure 13. Binding of CVB3 5′CL RNA constructs with CVB3 3C protein. The ITC profiles and corresponding binding curves for the WT 5′CL (a), 5′CL2 (b), and 5′CL4 (c) RNA constructs. The ITC measurements were conducted in the MicroCal PEAQ-ITC (Malvern Panalytical), and data were analyzed using its integrated software. The 19 successive injections of 2 µl of the 3C protein (~ 400 µM) were made into the RNA (~10 µM) in the calorimetry cell (see Methods for experimental details). The reported K_ds are the average ± standard deviation values from three independent measurements.

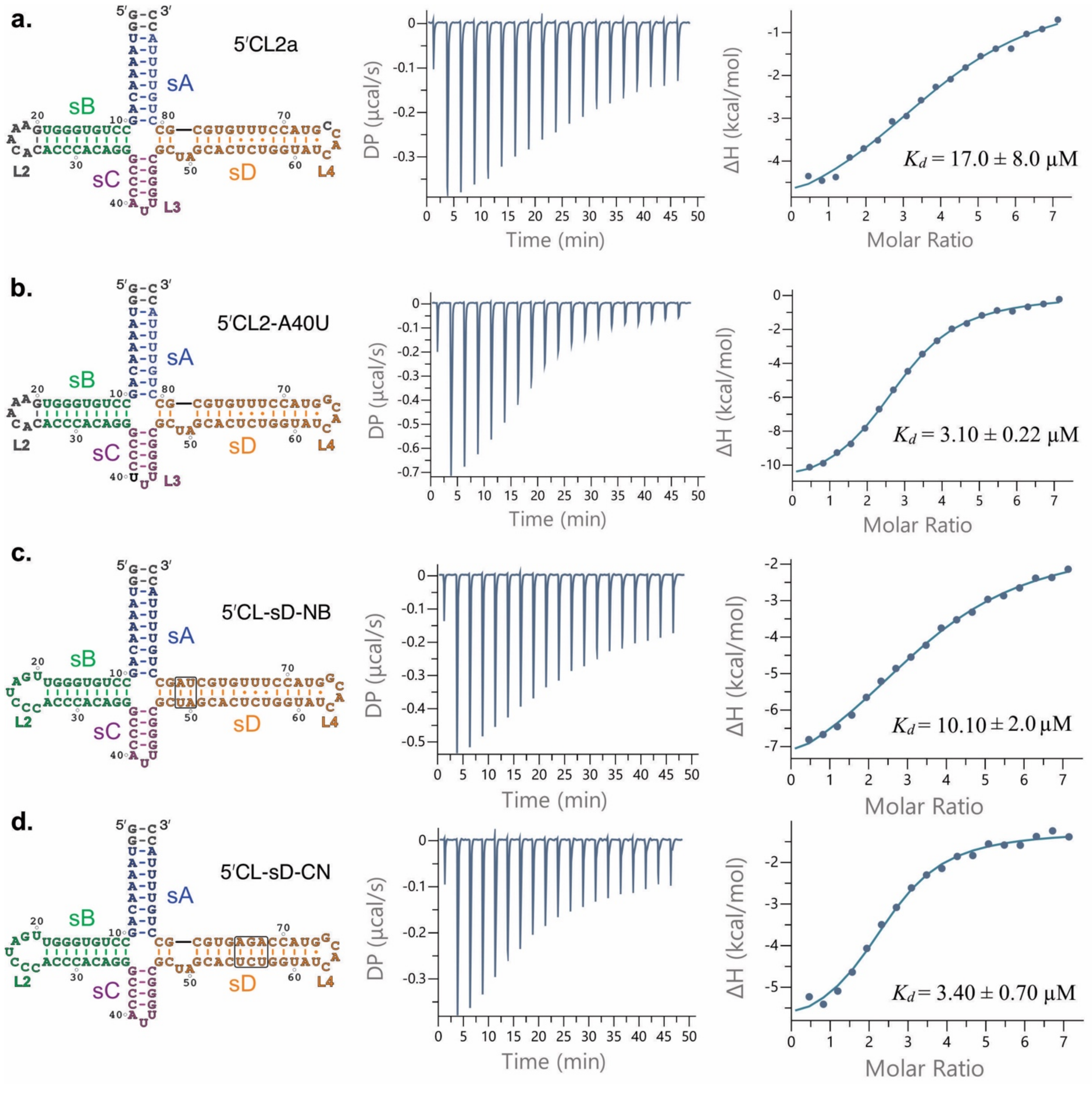

Supplementary Figure 14. Binding of CVB3 5′CL RNA constructs with CVB3 3C protein. The ITC profiles and corresponding binding curves for the 5′CL2a (a), 5′CL2-A40U (b), 5′CL-sD-NB (c), and 5′CL-sD-CN (d) RNA constructs. The ITC measurements were conducted in the MicroCal PEAQ-ITC (Malvern Panalytical), and data were analyzed using its integrated software. The 19 successive injections of 2 µl of the 3C protein (~ 400 µM) were made into the RNA (~10 µM) in the calorimetry cell (see Methods for experimental details). The reported K_ds are the average ± standard deviation values from three independent measurements.

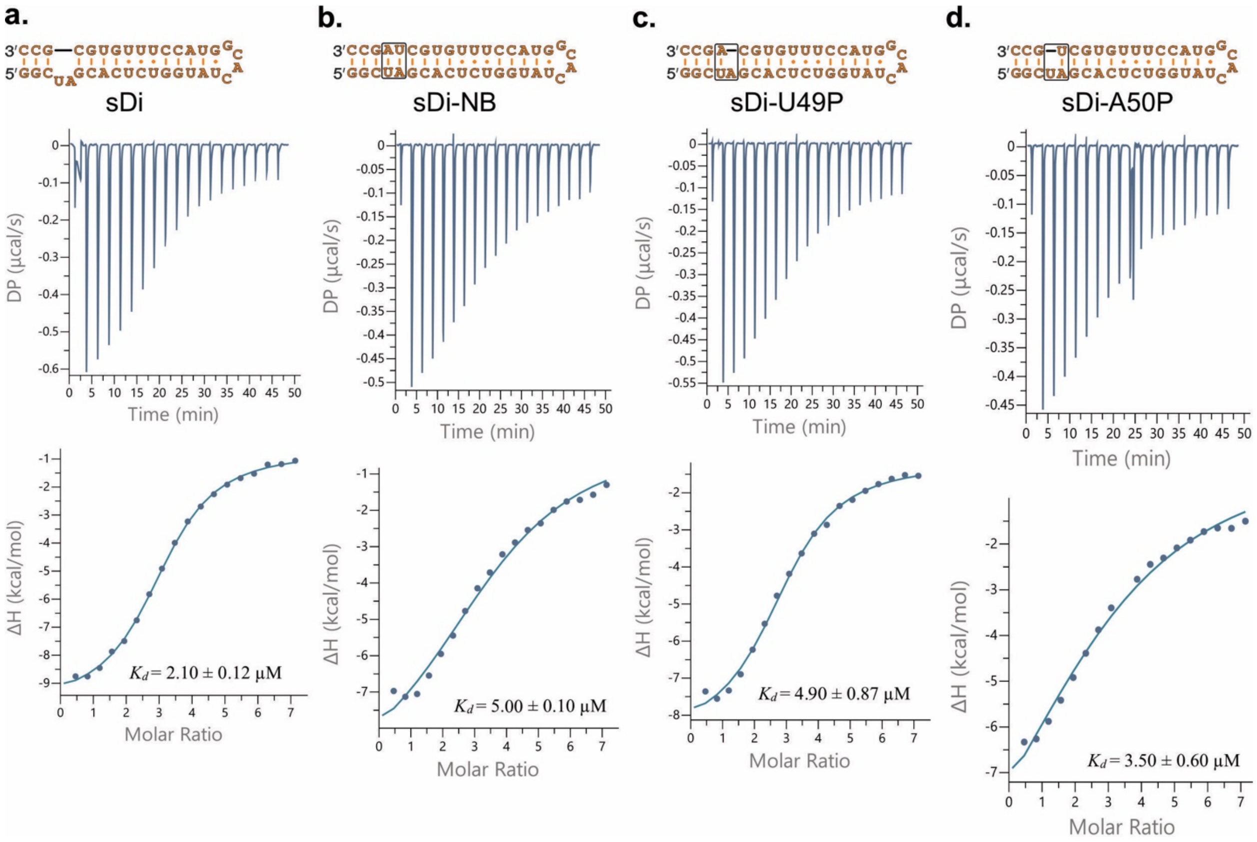

Supplementary Figure 15. Binding of CVB3 5′CL RNA constructs with CVB3 3C protein. The ITC profiles and corresponding binding curves for the isolated sD constructs, the sDi (a), sDi-NB (b), sDi-U49P (c), and sDi-A50P (d). The ITC measurements were conducted in the MicroCal PEAQ-ITC (Malvern Panalytical), and data were analyzed using its integrated software. The 19 successive injections of 2 µl of the 3C protein (~ 400 µM) were made into the RNA (~10 µM) in the calorimetry cell (see Methods for experimental details). The reported K_ds are the average ± standard deviation values from three independent measurements.

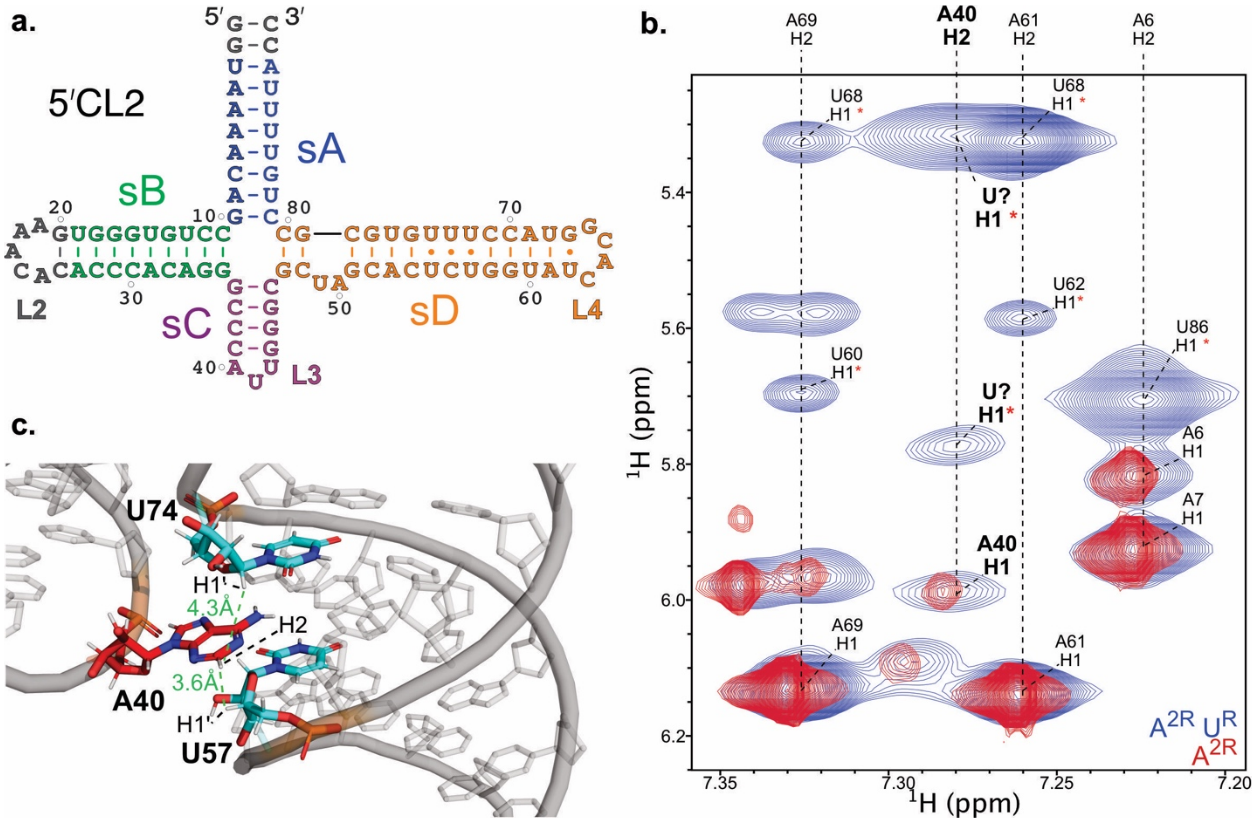

Supplementary Figure 16. The NMR studies of CVB3 5′CL2 construct to probe in solution the docking A40 into the Py-Py region. (a) The predicted secondary structure of the 5′CL2 RNA construct. (b) The NOE spectra of $A^{2R}U^{6R}$ (blue) and A^{2R} (red) labeled 5′CL2 constructs (see methods for the experimental details). The Uracil NOE signals highlighted with a star (*) were further cross-validated to arise from Uracils by U^{6R} labeled samples. (c) The crystal structure showing the interactions of A40 with U57 and U74, which is consistent with the NOE signals observed in solution NMR as shown in b. All H2 of all Adenines were confidentially assigned for the NOE data, except for the ones that only see the Uracils in the cross-strand and the ones in the Fab binding loop. Further studies are required to unambiguously rule out that the two Uracil signals from H2 A40 are from the triloop. The other NOE signals in this region (when following the peaks along the strand) get very broad, potentially due to the intrinsic flexibility within the region.

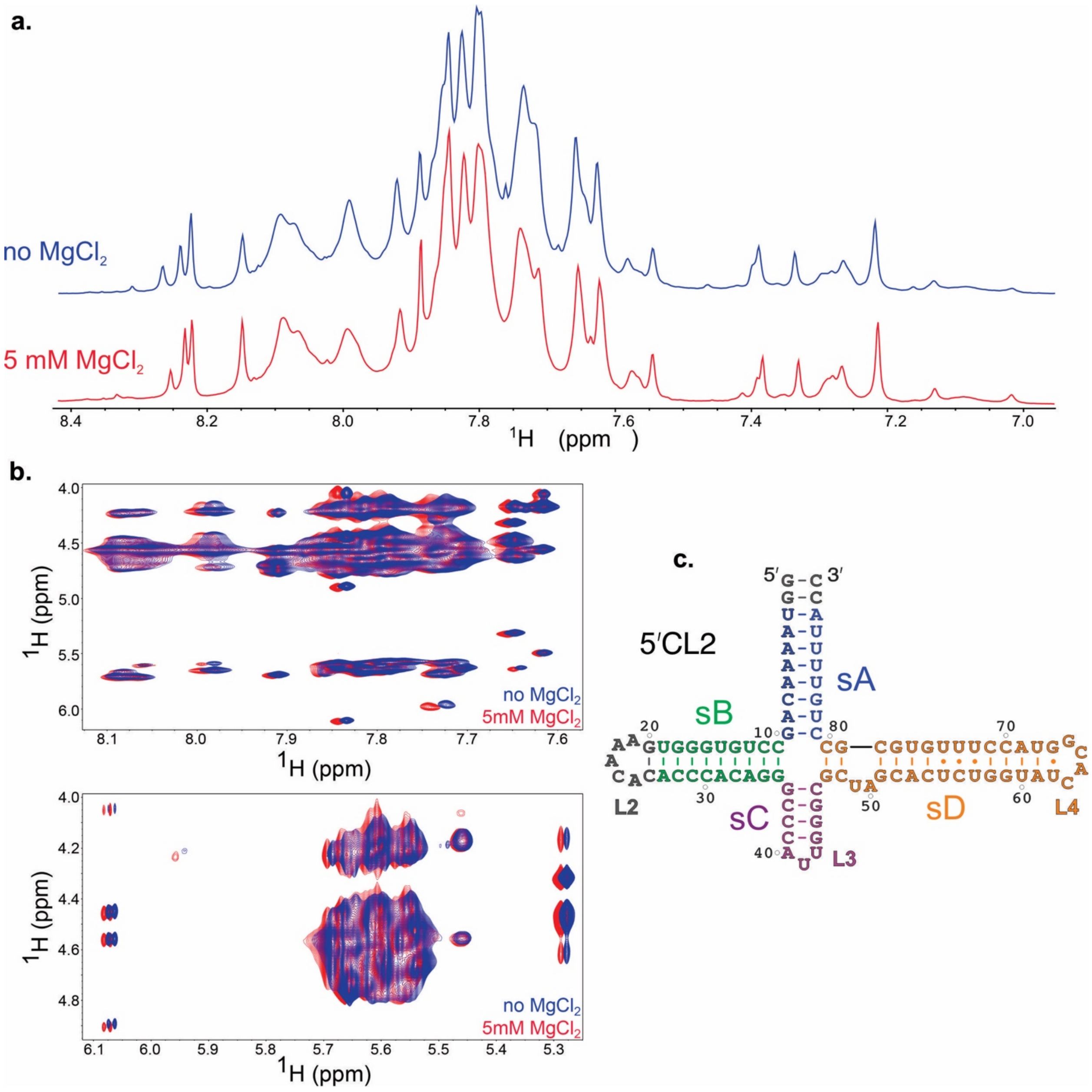

Supplementary Figure 17. The Mg^{2+} dependent NMR spectra of CVB3 5′CL2 construct. The 1D (a) and 2D (b) NOESY NMR spectrum of a U^{6R} labeled 5′CL2 RNA (c) in a buffer containing 20 mM sodium phosphate, pH 7.4, 70 mM KCl, 5 mM NaCl, and 5 mM $MgCl_2$ (red). The spectrum of the same sample after dialysis for 18 hours against the same buffer without the $MgCl_2$ (blue). The Y-axis in a has been offset for clarity.

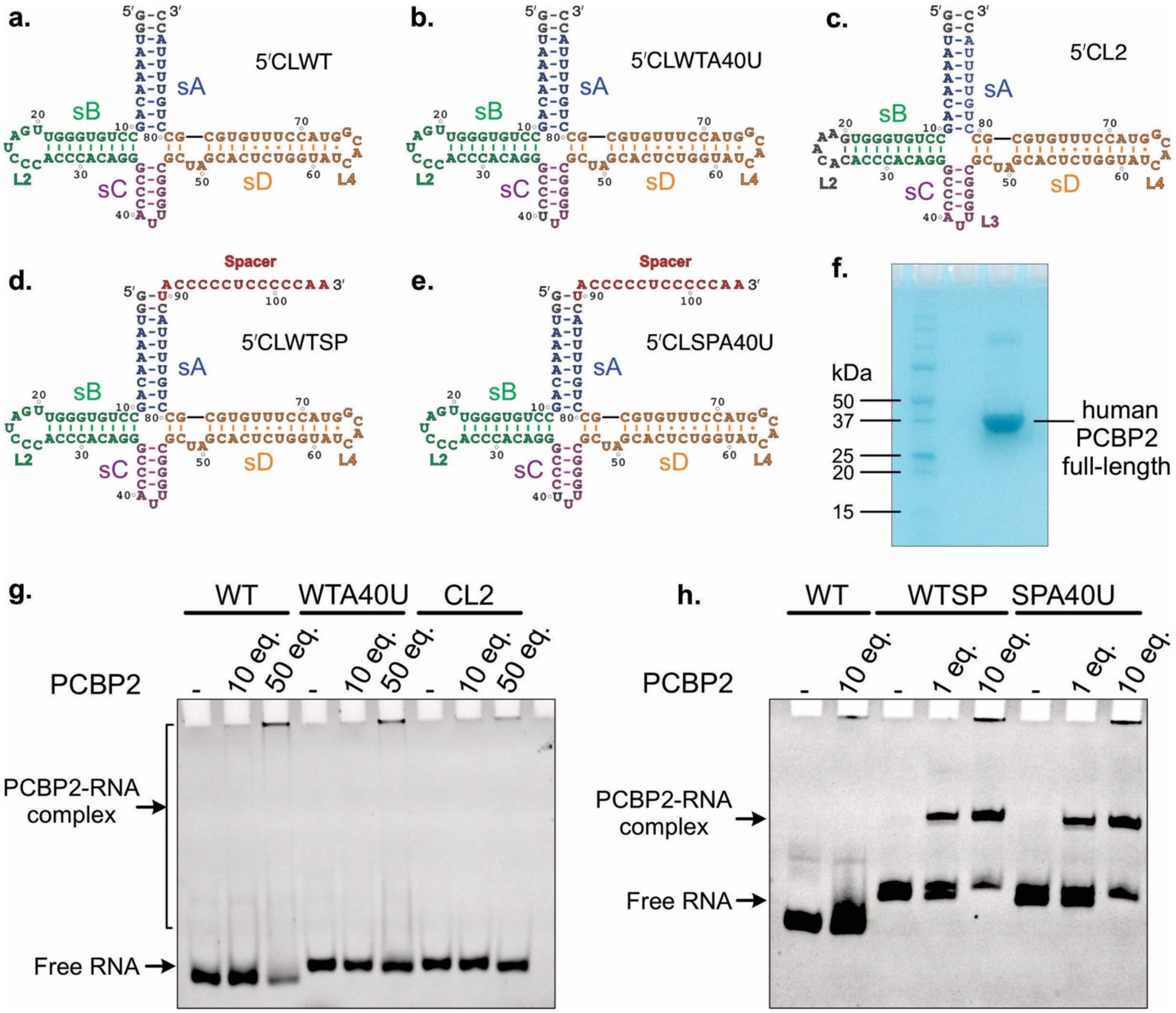

Supplementary Figure 18. The human PCBP2 binding tests for the 5′CLWT (a), 5′CLWTA40U (b), 5′CL2 (c), 5′CLWTSP (d), and 5′CLSPA40U (e) (see Methods for the experimental details). An SDS PAGE gel (f) showing the expected length and high purity of the recombinantly expressed human PCBP2 protein (see Methods for the expression and purification details). The nPAGE for testing the binding of PCBP2 with the core CVB3 5′CL constructs as shown in a-c (g) and with the longer constructs as shown in d and e (h). Each lane contains 100 ng of RNA. Although we did not observe a clear RNA-PCBP2 complex band for the core constructs, perhaps due to the weak affinity of PCBP2 for the sB loop, a PCBP2 dose-dependent shift of the RNA bands indicated the complex formation. Nevertheless, the longer RNA constructs with the 5′CL core plus the C-rich spacer sequence showed sharp, slow-migrating PCBP2-RNA complex bands in a PCBP2 dose-dependent manner, suggesting strong binding. Detailed studies with ITC are underway.

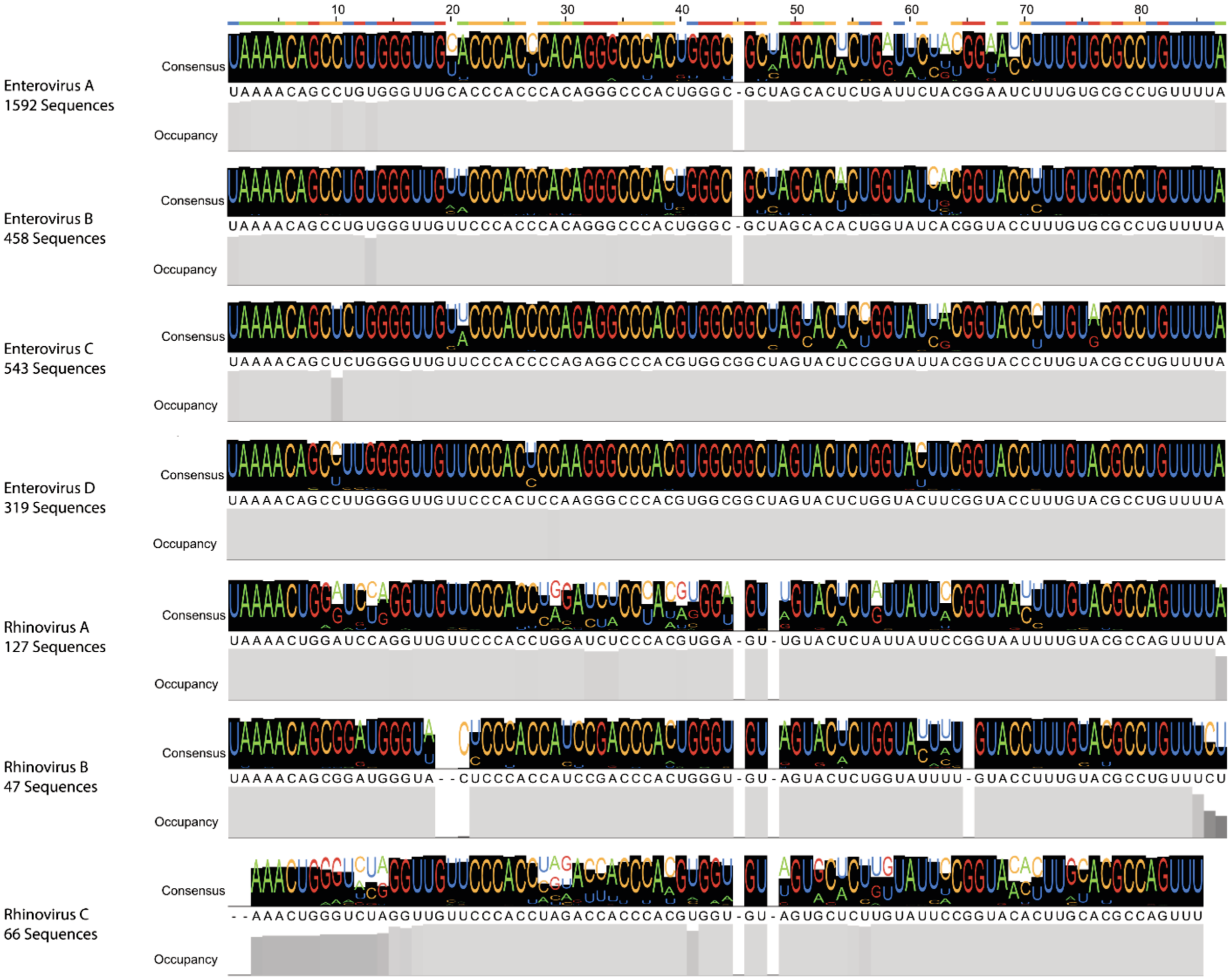

Supplementary Figure 19. The consensus sequences for the 7 types of human enterovirus species belonging to the enterovirus genus. The sequences available in the NCBI database nucleotide collection (nt/rt) (https://blast.ncbi.nlm.nih.gov/) for human enteroviral species were filtered for coverage completeness of the 5′CL region within the 5′UTR. These seed sequence data were then aligned using MuscleWS (http://www.compbio.dundee.ac.uk/jabaws/)[17] and visualized in Jalview (https://www.jalview.org/) software.[18] The alignment results show the nucleotide consensus and occupancy level within these 7 species, suggesting a high degree of sequence conservation within the 5′CL region of enteroviral genomes.

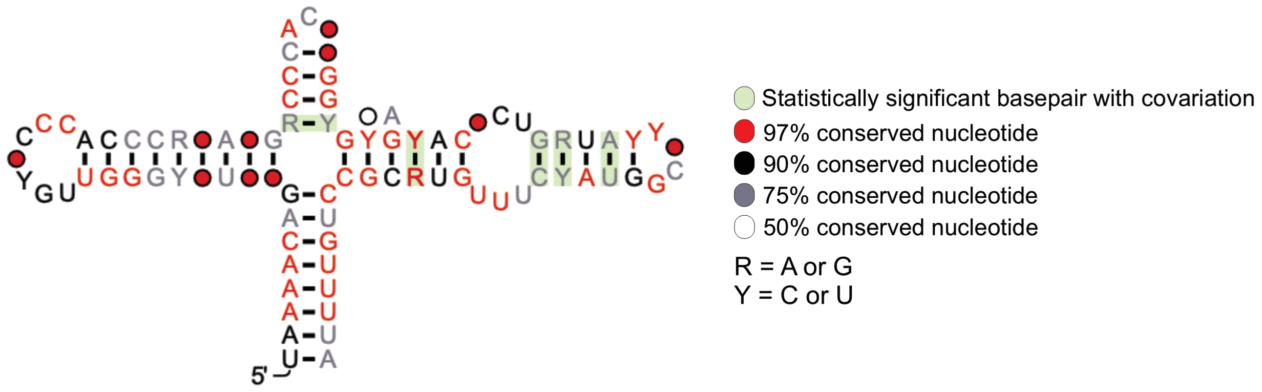

Supplementary Figure 20. The R-Scape (RNA Structural Covariation Above Phylogenetic Expectation) analysis (http://eddylab.org/R-scape/)[19] of the enteroviral 5′CL consensus secondary structure. As shown in Supplementary Figure 19 above, the 5′CL sequence alignments for all 7 enteroviral species were further refined to remove duplicate sequences using FASTX-Toolkit (http://hannonlab.cshl.edu/fastx_toolkit/), and the top 1000 most complete sequences were aligned via MuscleWS (http://www.compbio.dundee.ac.uk/jabaws/).[17] These aligned sequences were then uploaded into the R-Scape[19] to compute a consensus secondary structure of the enteroviral 5′CL. The secondary structure analysis indicates that the sequence and the structural features within each subdomain sA, sB, sC, and sD are highly conserved among known enteroviral species.

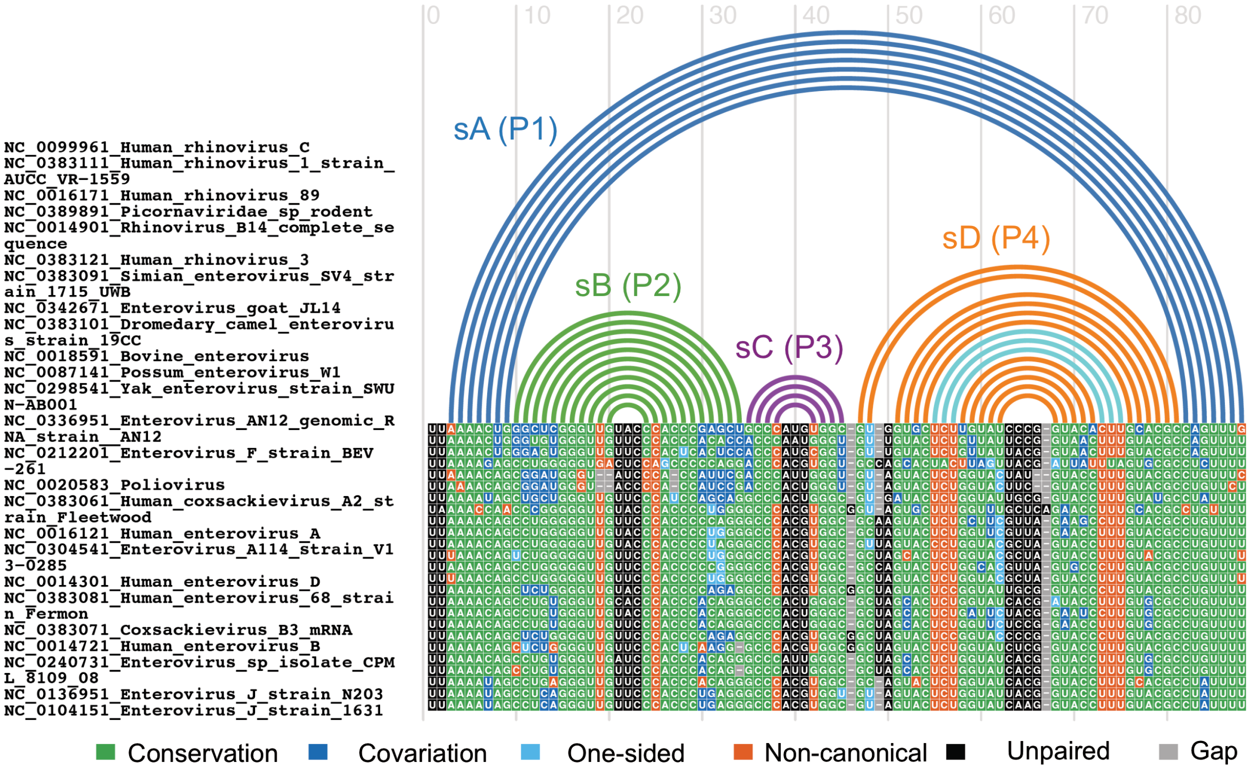

Supplementary Figure 21. The sequence alignment and base-pair conservation for the enteroviral 5′CL sequences. The alignment of the 25 reference enteroviral sequences available in the NCBI RefSeq database (https://www.ncbi.nlm.nih.gov/refseq/) with complete 5′CL sequences was performed using NCBI Nucleotide BLAST tool (https://blast.ncbi.nlm.nih.gov/Blast.cgi). The resulting multi-alignment file was visualized using the R-Chie (RNA Arc Diagram web server, https://www.e-rna.org/r-chie/).[20] The results further elucidate the high conservation of the base pair numbers within 5′CL subdomain helices and their alignment among all members of the enterovirus genus. Specifically, the length of the sC helix (4 base pairs) and sD helix preceding the Py-Py region (6 base pairs interrupted by dinucleotide bulge) appear highly conserved, which is consistent with the viral requirement for maintaining the long-range interactions between the sC A40 and the central C•U pair in the sD Py-Py helix as observed in our CVB3 5′CL crystal structure.

Supplementary information references

1. Bailey, J.M. & Tapprich, W.E. Structure of the 5' nontranslated region of the coxsackievirus B3 genome: Chemical modification and comparative sequence analysis. *J Virol* **81**, 650-68 (2007).
2. Das, R., Karanicolas, J. & Baker, D. Atomic accuracy in predicting and designing noncanonical RNA structure. *Nature methods* **7**, 291 (2010).
3. Lyskov, S. et al. Serverification of Molecular Modeling Applications: The Rosetta Online Server That Includes Everyone (ROSIE). *PLOS ONE* **8**, e63906 (2013).
4. Du, Z., Yu, J., Andino, R. & James, T.L. Extending the Family of UNCG-like Tetraloop Motifs: NMR Structure of a CACG Tetraloop from Coxsackievirus B3. *Biochemistry* **42**, 4373-4383 (2003).
5. Du, Z., Yu, J., Ulyanov, N.B., Andino, R. & James, T.L. Solution structure of a consensus stem-loop D RNA domain that plays important roles in regulating translation and replication in enteroviruses and rhinoviruses. *Biochemistry* **43**, 11959-72 (2004).
6. Ohlenschläger, O. et al. The structure of the stemloop D subdomain of coxsackievirus B3 cloverleaf RNA and its interaction with the proteinase 3C. *Structure* **12**, 237-48 (2004).
7. Prusa, J., Missak, J., Kittrell, J., Evans, J.J. & Tapprich, W.E. Major alteration in coxsackievirus B3 genomic RNA structure distinguishes a virulent strain from an avirulent strain. *Nucleic Acids Research* **42**, 10112-10121 (2014).
8. Mahmud, B., Horn, C.M. & Tapprich, W.E. Structure of the 5' Untranslated Region of Enteroviral Genomic RNA. *J Virol* **93**(2019).
9. Warden, M.S. et al. Conformational flexibility in the enterovirus RNA replication platform. *RNA* **25**, 376-387 (2019).
10. Sharma, N. et al. Functional role of the 5′ terminal cloverleaf in Coxsackievirus RNA replication. *Virology* **393**, 238-249 (2009).
11. Zell, R., Sidigi, K., Bucci, E., Stelzner, A. & Görlach, M. Determinants of the recognition of enteroviral cloverleaf RNA by coxsackievirus B3 proteinase 3C. *RNA (New York, N.Y.)* **8**, 188-201 (2002).

12. Roehl, H.H. & Semler, B.L. Poliovirus infection enhances the formation of two ribonucleoprotein complexes at the 3' end of viral negative-strand RNA. *Journal of Virology* **69**, 2954-2961 (1995).
13. Banerjee, R., Echeverri, A. & Dasgupta, A. Poliovirus-encoded 2C polypeptide specifically binds to the 3'-terminal sequences of viral negative-strand RNA. *Journal of Virology* **71**, 9570-9578 (1997).
14. Banerjee, R., Tsai, W., Kim, W. & Dasgupta, A. Interaction of Poliovirus-Encoded 2C/2BC Polypeptides with the 3′ Terminus Negative-Strand Cloverleaf Requires an Intact Stem-Loop b. *Virology* **280**, 41-51 (2001).
15. Brunner, J.E. et al. Functional Interaction of Heterogeneous Nuclear Ribonucleoprotein C with Poliovirus RNA Synthesis Initiation Complexes. *Journal of Virology* **79**, 3254-3266 (2005).
16. Ertel, K.J., Brunner, J.E. & Semler, B.L. Mechanistic consequences of hnRNP C binding to both RNA termini of poliovirus negative-strand RNA intermediates. *J Virol* **84**, 4229-42 (2010).
17. Edgar, R.C. Muscle5: High-accuracy alignment ensembles enable unbiased assessments of sequence homology and phylogeny. *Nature Communications* **13**, 6968 (2022).
18. Waterhouse, A.M., Procter, J.B., Martin, D.M.A., Clamp, M. & Barton, G.J. Jalview Version 2 − a multiple sequence alignment editor and analysis workbench. *Bioinformatics* **25**, 1189-1191 (2009).
19. Rivas, E., Clements, J. & Eddy, S.R. A statistical test for conserved RNA structure shows lack of evidence for structure in lncRNAs. *Nature Methods* **14**, 45-48 (2017).
20. Lai, D., Proctor, J.R., Zhu, J.Y.A. & Meyer, I.M. R- chie : a web server and R package for visualizing RNA secondary structures. *Nucleic Acids Research* **40**, e95-e95 (2012).
