# Supplementary material for: Crystal structure of a highly conserved enteroviral 5′ cloverleaf RNA replication element
Source: Nat Commun. 2023 Apr 7;14:1955. doi: 10.1038/s41467-023-37658-8 (PMC10082201; doi:10.1038/s41467-023-37658-8)
Supplement: Supplementary file 3 — Reporting Summary [file 41467_2023_37658_MOESM3_ESM.pdf]

nature portfolio

Corresponding author(s): Deepak Koirala

Last updated by author(s): Mar 8, 2023

Reporting Summary

Nature Portfolio wishes to improve the reproducibility of the work that we publish. This form provides structure for consistency and transparency in reporting. For further information on Nature Portfolio policies, see our Editorial Policies and the Editorial Policy Checklist.

Statistics

For all statistical analyses, confirm that the following items are present in the figure legend, table legend, main text, or Methods section.

n/a	Confirmed	
☐	☒	The exact sample size (*n*) for each experimental group/condition, given as a discrete number and unit of measurement
☐	☒	A statement on whether measurements were taken from distinct samples or whether the same sample was measured repeatedly
☒	☐	The statistical test(s) used AND whether they are one- or two-sided<br>*Only common tests should be described solely by name; describe more complex techniques in the Methods section.*
☒	☐	A description of all covariates tested
☐	☐	A description of any assumptions or corrections, such as tests of normality and adjustment for multiple comparisons
☐	☒	A full description of the statistical parameters including central tendency (e.g. means) or other basic estimates (e.g. regression coefficient) AND variation (e.g. standard deviation) or associated estimates of uncertainty (e.g. confidence intervals)
☒	☐	For null hypothesis testing, the test statistic (e.g. *F*, *t*, *r*) with confidence intervals, effect sizes, degrees of freedom and *P* value noted<br>*Give P values as exact values whenever suitable.*
☒	☐	For Bayesian analysis, information on the choice of priors and Markov chain Monte Carlo settings
☒	☐	For hierarchical and complex designs, identification of the appropriate level for tests and full reporting of outcomes
☒	☐	Estimates of effect sizes (e.g. Cohen's *d*, Pearson's *r*), indicating how they were calculated

Our web collection on statistics for biologists contains articles on many of the points above.

Software and code

Policy information about availability of computer code

Data collection	A detailed description of all data collection strategies is provided in the methods section. X-ray crystallographic data were collected at the Argonne National Laboratory beam-lines 24-ID-C and 24-ID-E. The ITC data were collected using MicroCal PEAQ-ITC (Malvern Panalytical Ltd) equipment.
Data analysis	A detailed description of data analysis and related software is provided in the methods section. The crystallographic data analysis were performed using Phenix 1.20.1-4487 and COOT 0.9.8.1 software. The ITC data were analyzed using MicroCal PEAQ-ITC software.

For manuscripts utilizing custom algorithms or software that are central to the research but not yet described in published literature, software must be made available to editors and reviewers. We strongly encourage code deposition in a community repository (e.g. GitHub). See the Nature Portfolio guidelines for submitting code & software for further information.

Data

Policy information about availability of data

All manuscripts must include a data availability statement. This statement should provide the following information, where applicable:

- Accession codes, unique identifiers, or web links for publicly available datasets
- A description of any restrictions on data availability
- For clinical datasets or third party data, please ensure that the statement adheres to our policy

The atomic coordinates and structure factors for the reported crystal structure have been deposited with the Protein Data Bank under accession code 8DP3.

Authors will provide the raw data, additional information, and materials, including the plasmid for Fab BL3-6 expression, upon reasonable request. These include, but not limited to, the expression plasmids for Fab BL3-6, 3C, and PCBP2 proteins. The requests should be addressed to Deepak Koirala.

Human research participants

Policy information about studies involving human research participants and Sex and Gender in Research.

Reporting on sex and gender	NA
Population characteristics	NA
Recruitment	NA
Ethics oversight	NA

Note that full information on the approval of the study protocol must also be provided in the manuscript.

Field-specific reporting

Please select the one below that is the best fit for your research. If you are not sure, read the appropriate sections before making your selection.

☒ Life sciences ☐ Behavioural & social sciences ☐ Ecological, evolutionary & environmental sciences

For a reference copy of the document with all sections, see nature.com/documents/nr-reporting-summary-flat.pdf

Life sciences study design

All studies must disclose on these points even when the disclosure is negative.

Sample size	The sample size chosen for each experiment (n=3) were sufficient to support the conclusions. Sample sizes are indicated within the figure legends wherever applicable. No statistical methods were used to determine the sample size.
Data exclusions	No data was excluded from the analysis except for the failed experiments. The reflection data outliers in the crystallographic dataset were automatically excluded using the established programs and criteria (I/σI > 1 and CC1/2 > 0.3).
Replication	All attempts to reproduce the reported datasets were successful. The experiments, unless specified otherwise, were performed in triplicate.
Randomization	Randomization is not applicable as experiments did not involved any organisms or sample groups.
Blinding	No author was aware of the outcomes of the experiments.

Reporting for specific materials, systems and methods

We require information from authors about some types of materials, experimental systems and methods used in many studies. Here, indicate whether each material, system or method listed is relevant to your study. If you are not sure if a list item applies to your research, read the appropriate section before selecting a response.

Materials & experimental systems

n/a	Involved in the study	
☐	☒	Antibodies
☒	☐	Eukaryotic cell lines
☒	☐	Palaeontology and archaeology
☒	☐	Animals and other organisms
☒	☐	Clinical data
☒	☐	Dual use research of concern

Methods

n/a	Involved in the study	
☒	☐	ChIP-seq
☒	☐	Flow cytometry
☒	☐	MRI-based neuroimaging

Antibodies

Antibodies used	This research involved synthetic anti-RNA antibody (Fab) that was selected in vitro through a phage display selection method.
Validation	A detailed description about the Fab antibody used in this study is provided in the methods section.
